# Local Anesthesia, Infiltrative, Conductive and Intraosseous Methods

**Published:** 1918-03

**Authors:** George N. Hein

**Affiliations:** San Francisco, Cal.


### LOCAL ANESTHESIA, INFILTRATIVE, CONDUCTIVE AND INTRAOSSEOUS METHODS

BY GEORGE N. HEIN, SAN FRANCISCO, CAL.

Anesthetic used, two per cent. novacain, 1/30000 Suprarenin.

CASE ONE

Patient, young woman aged twenty; operation, preparation of upper left bicuspid for jacket crown.

The field of operation being cleansed by means of the atomizer and thoroughly bathed with a seventy per cent. solution of alcohol, cotton rolls are so placed as to protect it from the saliva and a pin point application of phenol is made between the molar and bicuspid to render the puncture with the needle painless.

A few drops of the anesthetic solution was then injected into the interdental flap by means of a peridental syringe mounted with a one-quarter inch No. 28 gauge needle directed toward the bucco-apical region of the tooth to be anesthetized. This needle was then replaced with a conical needle having a point about the size of a number one-half rose head bur and the syringe placed in a convenient position for future use.

The lips, cheek and membrane were then held under tension and an incision one-sixteenth of an inch long made with a lancet through the gum between the upper thirds of the roots of the molar and bicuspid.

With a number one-half round bur in the engine handpiece the outer plate of bone was punctured where previously exposed by the incision, the entrance into the cancellous tissue being denoted by a sensation similar to that felt when opening into the pulp chamber. The conical needle was then inserted into the opening made by the bur and twenty minims of the anesthetic injected, three minutes being consumed in the operation to avoid undue stimulation, as in this method direct venal injections are made. The tooth was then painlessly prepared for the crown in the usual manner; time consumed one hour and fifteen minutes.

CASE TWO

Patient, man, aged forty-five; operation, extraction of ten upper anterior teeth, five being vital.

The region to be operated upon was prepared as in the previous case and the syringe fitted with the one-quarter inch No. 28 gauge needle. Four intraosseous injections were made by gently tapping the needle through the outer plate of bone into the cancellous tissue between the bicuspids, and the cuspids and laterals on each side, the puncture being made through the interdental flaps slightly above the gingival border of the teeth. Five minutes were consumed in making the injections during which sixty minims of the anesthetic were administered. The pulps of the five vital teeth were then entered with a bur to prove their complete anesthesia. The teeth were then extracted without pain.

CASE THREE

Patient, man aged thirty-five; operation, extraction of upper right third molar.

The region to be operated upon was prepared as in the previous case and the needle tapped through the outer plate of bone into the cancellous tissue, the puncture being made through the interdental papilla between the second and third molars. Anesthesia was obtained and the tooth extracted in four minutes without discomfort to the patient.

CASE FOUR

Patient, man aged thirty; operation, extraction of impacted lower third molar.

Preparation made as in previous cases and conductive anesthesia employed by making the pterigo-mandibular injection.

Twenty-five minims of the anesthetic were injected in the region of the mandibular foramen and as results by this method are not obtained within fifteen or twenty minutes, mucous anesthesia was also employed, an injection being made on each side of the tooth to be extracted. By this means the operator was enabled to commence operations on the overlying soft tissues and also remove whatever osseous tissue may have overlain the tooth. The operation was commenced immediately and the tooth removed.

The operation consumed one hour and twenty minutes, during which there was complete anesthesia.—Panama-Pacific Dental Congress Clinic as reported in *Dental Items of Interest* for November.

